# Diagnostic and Therapeutic Approach to Metachronous Splenic Metastases of Gastric Adenocarcinoma: Case Report and Literature Review

**DOI:** 10.3390/diagnostics15202570

**Published:** 2025-10-12

**Authors:** Cosmina Fugărețu, Sandu Ramboiu, Cătălin Mișarca, Corina Maria Dochit, Mihail Virgil Boldeanu, Stefan Patrascu, Valeriu Șurlin

**Affiliations:** 1 Doctoral School, University of Medicine and Pharmacy of Craiova, 200349 Craiova, Romania; comanescu_cosmina@yahoo.com (C.F.); vsurlin@gmail.com (V.Ș.); 2 1st General Surgery Department, Brașov County Emergency Clinical Hospital, 500326 Brașov, Romania; 3 Faculty of General Medicine Brașov, Transilvania University, 500036 Brașov, Romania; 4 1st Clinic of Surgery, Emergency Hospital of Craiova, 200642 Craiova, Romania; stef.patrascu@gmail.com; 5 Faculty of General Medicine Craiova, University of Medicine and Pharmacy of Craiova, 200642 Craiova, Romania; 6 Department of Pathology, Brașov County Emergency Clinical Hospital, 500326 Brașov, Romania; dochitcorina@yahoo.com; 7 Department of Immunology, University of Medicine and Pharmacy of Craiova, 2 Petru Rares Street, 200349 Craiova, Romania; boldeanumihailvirgil@yahoo.com

**Keywords:** gastric cancer, splenic metastases, Dickkopf Related Protein 3-DKK3, Cluster of Differentiation 276-CD 276

## Abstract

**Background and Clinical Significance**: In gastric cancer, splenic metastases are found in less than 7% of cases and are usually associated with other systemic secondary determinations; much more rarely, they represent the sole secondary determination of the malignant disease. **Case presentation**: In this paper, we present the case of a 64-year-old patient who underwent curative surgery for gastric adenocarcinoma 10 months ago and, during oncological monitoring, was diagnosed with a splenic tumor formation with intense metabolic activity on PET-CT examination, raising suspicion of splenic metastases. The medical team observed an increase in carcinoembryonic antigen (CEA), carbohydrate antigen (CA) 19-9, and Cluster of Differentiation (CD) 276 values, along with a slight decrease in Dickkopf Related Protein 3 (DKK 3). Considering that the spleen was the only site of secondary localization of the malignant disease, the patient underwent laparoscopic splenectomy with histopathological confirmation of the presence of gastric adenocarcinoma. There are no signs of loco-regional or distant recurrence 15 months postoperatively. In patients with radical excision of gastric cancer who present only with splenic metastases, splenectomy is indicated and is associated with good disease-free survival. If other secondary manifestations of malignant gastric disease are identified or suspected, chemotherapy treatment and the wait-and-see approach are recommended, as the patient does not have a real benefit from splenectomy. Until now, there is no standard protocol for the diagnostic and therapeutic management of patients with gastric cancer and metachronous splenic metastases; thus, the development of a decision-making scheme for these situations is necessary. **Conclusions**: The multidisciplinary approach, including the tumor board and an infectious disease specialist, are important steps in the effective management of these cases. The role of new biological markers such as CD 276 and DKK 3 for assessing the progression of malignant disease could constitute a new direction for research.

## 1. Introduction

The incidence of splenic metastases in patients with malignant diseases is low, ranging between 0.6% and 1.1%, and among patients with gastric cancer, it is estimated to be just under 7% of cases [1,2].

Most frequently, secondary splenic lesions of solid tumors are encountered in the context of multiorgan spread of malignant disease and are much rarer as solitary lesions [3].

Some studies suggest that malignant melanoma is the most common solitary tumor that can be associated with splenic metastases, while other studies identify lung cancer as the primary site of the primary tumor [1,3,4]. There are also documented cases of breast, ovarian, colorectal, and gastric cancers presenting with splenic parenchymal metastases [1,3,4].

In over 60% of cases, occurrences of secondary metachronal splenic determinations are asymptomatic and are discovered during the imaging monitoring of neoplastic patients; splenomegaly can raise clinical suspicion, as fever and fatigue are symptoms that may occur in gastric cancer [3,5]. Dynamic increases in carcinoembryonic antigen (CEA) and carbohydrate antigen (CA) 19-9 values have also been observed in patients with a history of gastric cancer who present with splenic metachronal metastases [6,7]. Although new markers such as Human Dickkopf Related Protein 3 (DKK3) and Human Cluster of Differentiation 276 (CD 276) have been discovered, which may be useful in assessing the prognosis of gastric cancer patients, their role in postoperative follow-up is not yet elucidated.

It seems that in gastric cancer, elevated serum levels of CD 276 are associated with a negative prognosis of the malignant disease and positively correlate with tumor stage, tumor differentiation, and depth of tumor extension; they also negatively correlate with overall survival rate [8]. On the other hand, low serum levels of DKK3 are correlated with a more advanced tumor stage, the presence of lymph node invasion, perivascular and perineural invasion, and a lower overall survival rate [9].

In this paper, we present a rare case of metachronous splenic metastasis of a gastric adenocarcinoma that underwent a radical gastrectomy with D2 lymphadenectomy 10 months earlier, which benefited from laparoscopic splenectomy followed by a favorable course, with no recurrence of the malignant disease at 15 months postoperatively. We also conducted a review of the specialized literature with the aim of developing a diagnostic and therapeutic approach scheme for patients with gastric cancer who present with metachronous splenic metastases.

## 2. Case Presentation

We present the case of a 64-year-old patient known to have stage IIIB antral gastric cancer according to the American Joint Committee on Cancer (AJCC) 8th Edition (2018+) and presenting a positive status for Helicobacter pylori [10]. Initially, the patient underwent 3 cycles of neoadjuvant chemotherapy using the FLOT regimen, which includes 5-fluorouracil, docetaxel, oxaliplatin, and calcium folinate. Six weeks after completing chemotherapy, he had a laparoscopic subtotal gastrectomy with D2 lymphadenectomy, without resection of station 10 lymph nodes. Preoperatively, 5 mL of venous blood were collected, and human DKK3 and human CD 276 were determined using the Enzyme Linked Immunosorbent Assay Kit (ELISA Kit, Elabscience, Houston, TX, USA), with the aim of enrolling the patient in a larger study to assess the prognostic role of the aforementioned markers in gastric cancer. Thus, the CD 276 value was 90.310 ng/mL (sensitivity 0.47 ng/mL, detection range 0.78–50 ng/mL), which is above the average value recorded in cancer-free individuals, which is 34.894, and the DKK3 value was 84.646 ng/mL, below the average DKK3 value of 110.57 ng/mL detected in healthy individuals (sensitivity 0.47 ng/mL, detection range 0.78–50 ng/mL).

Initially, the determination of CEA and CA19-9 values was performed at the time of diagnosis when a CA value of 6.8 ng/mL (normal < 3 ng/mL for non-smokers, <5 ng/mL for smokers) and a CA19-9 value of 108 U/mL (normal < 27 U/mL) were observed. The dynamic evolution of the previously mentioned tumor markers is specified in Table 1 and Table 2.

**Figure 1:**
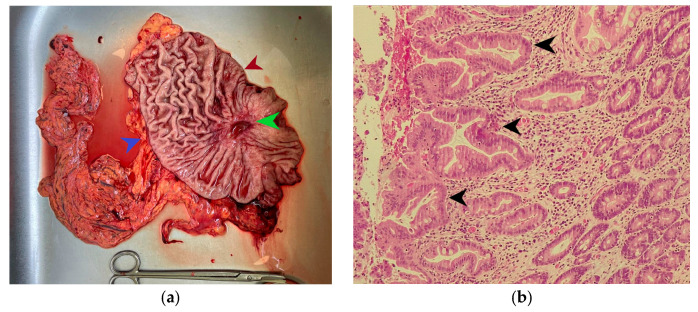
(**a**) Subtotal gastric resection specimen. The green arrow indicates an ulcerated gastric tumor measuring 3/2.5 cm, the red arrow indicates the lesser curvature of the stomach, and the blue arrow marks the greater curvature of the stomach. (**b**) Microscopic appearance of the gastric tumor in hematoxylin-eosin staining, magnification ×200; the black arrow indicates areas of moderately differentiated adenocarcinoma on an intensely desmoplastic background.

**Figure 2:**
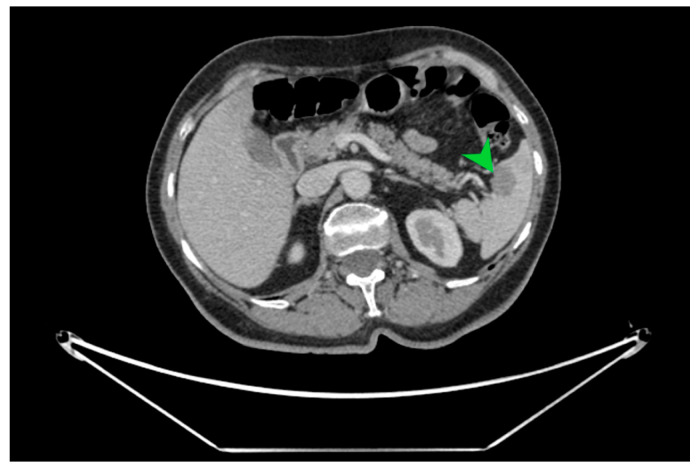
CT image in cross-section on which the well-defined hypodense splenic tumor formation of 23.5/16 mm is indicated with a green arrow, with a high suspicion of splenic metastasis.

**Figure 3:**
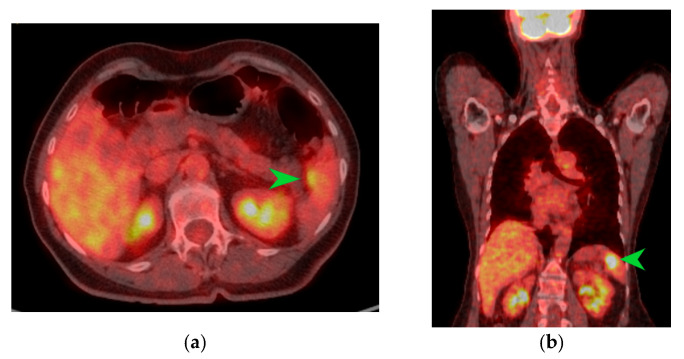
PET-CT images in transverse (**a**) and coronal (**b**) sections highlighting an area of metabolic activity in the spleen that raises suspicion of splenic metastasis. The splenic tumor is indicated by the green arrow.

The macroscopic examination of the gastric resection specimen revealed a tumor ulceration of 3/2.5 cm (Figure 1a). Microscopic evaluation confirmed the presence of moderately differentiated intestinal-type gastric adenocarcinoma (G2), infiltrative in the subserosa, with vascular tumor emboli present (Figure 1b). Twenty-eight lymph nodes were identified, and tumor invasion was confirmed in five of them. The surgical resection margins were free of tumor invasion. Postoperatively, the patient remained under oncological follow-up, and approximately 10 months postoperatively, he reported significant weight loss and physical asthenia, symptoms that were initially considered to be associated with the complex treatment of the neoplastic disease. However, serum determination showed a slight increase in CEA to 6.5 ng/mL from 3.2 ng/mL and an increase in CA19-9 to 160 U/mL from 21 U/mL (Table 1), which led to the decision to perform an abdominal CT that revealed the presence of a hypodense, poorly iodophilic splenic lesion, measuring 23.5/16 mm, raising suspicion of a splenic metachronous metastasis (Figure 2). The imaging examination was continued with a PET-CT scan, which highlighted the pathological uptake of 18F-2-deoxy-2-fluoro-glucose (18F-FDG) at the level of the previously described lesions without detecting other areas of suspected metabolic hyperactivity (Figure 3).

After obtaining the patient’s consent, it was decided to repeat the determination of CD 276 and DKK3, which revealed a slight increase in the former to 94.140 ng/mL and a decrease in the latter to 78.490 ng/mL.

A multidisciplinary tumor board discussion, considering that no other secondary determinations of the malignant disease were diagnosed radiologically, indicated a laparoscopy to exclude peritoneal carcinomatosis and splenectomy.

Twenty-one days before the surgery, the patient received pneumococcal, meningococcal, and Haemophilus influenzae type b vaccinations.

**Figure 4:**
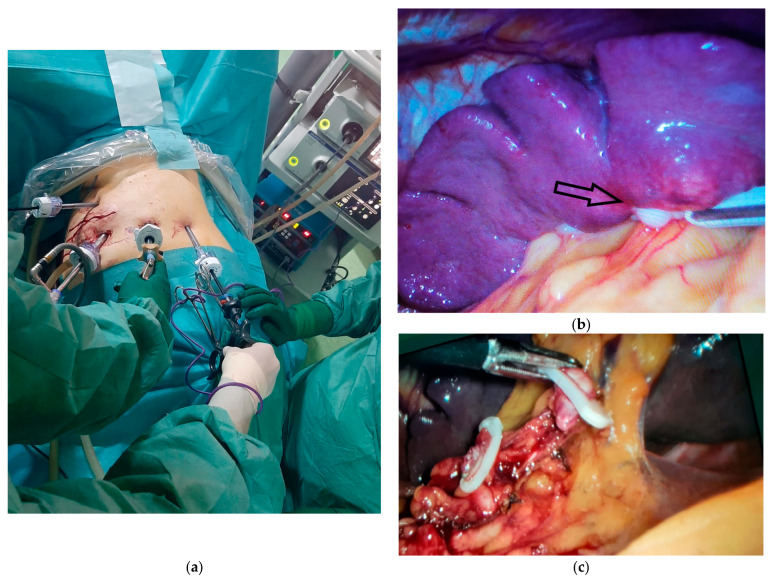
Intraoperative aspects of laparoscopic splenectomy. (**a**) Positioning the working trocars for performing the surgical procedure. (**b**) Intraoperative appearance of the splenic metastasis from gastric cancer, indicated by the black arrow. (**c**) The intraoperative appearance of the dissection of the splenic vessels at the hilar level with the elevation of lymph node station 10 and the preservation of 2 short gastric branches for the remaining stomach.

The peritoneal exploration was performed with the patient in the supine position and ruled out the presence of peritoneal carcinomatosis; however, splenic tumor lesions adjacent to the hilum were visualized (Figure 4b). For the easier performance of laparoscopic splenectomy, the patient was repositioned in the right lateral decubitus position with the left arm abducted at 90 degrees, and the working trocars were repositioned (Figure 4a).

The dissection of the splenic hilum was more difficult due to postoperative adhesions. The splenic artery was initially identified, clipped, and sectioned, followed by the clipping and sectioning of the splenic vein while preserving 2–3 short gastric vessels that ensure the vascularization of the remaining stomach (Figure 4c). The dissection of station 10 lymph nodes was performed, which had not been excised in the initial intervention.

The postoperative course was uneventful, followed by the appearance of thrombocytosis over 900,000 five days postoperatively, which is why anticoagulant treatment with Clexane was continued, along with the addition of an antiplatelet agent—aspirin 100 mg.

**Figure 5:**
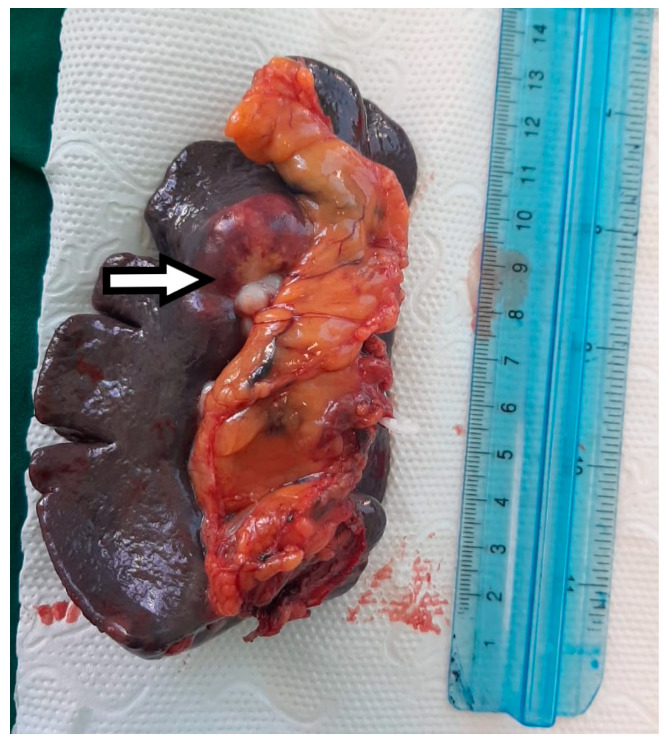
The macroscopic appearance of the splenectomy specimen with a hilar metastatic gastric cancer tumor indicated by the white arrow.

**Figure 6:**
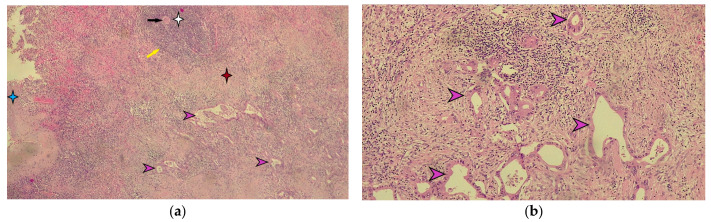
Microscopic appearance of splenic metastasis. (**a**) Microscopic image of the splenic metastasis of gastric cancer stained with hematoxylin-eosin (H-E), ×40 magnification. The blue star indicates the splenic capsule. The white star indicates the white pulp of the spleen, highlighting the central arteriole indicated by the black arrow and the marginal zone marked by the yellow arrow. The red star indicates the red pulp of the spleen. The purple arrows indicate the metastatic tumor of moderately differentiated gastric adenocarcinoma. (**b**) Detailed microscopic image of splenic metastasis of moderately differentiated gastric adenocarcinoma in hematoxylin-eosin staining indicated by purple arrows (magnification ×100).

Macroscopic histopathological examination of the resection specimen revealed a spleen measuring 15/10/4 cm with whitish solid tumors located at the hilum and subcapsular (Figure 5). Microscopically, splenic parenchyma with distorted architecture was identified due to areas of intestinal-type adenocarcinoma (Figure 6). No tumor invasion was observed in the station 10 lymph nodes.

Postoperatively, the patient underwent 3 cycles of chemotherapy with S-1 (Teysuno) and cisplatin. CEA and CA 19-9 values determined approximately 6 months postoperatively were within normal limits (Table 1).

The patient does not present loco-regional or distant recurrence 15 months postoperatively and is still under oncological follow-up.

## 3. Discussion

The literature rarely cites splenic metastases of gastric cancer, despite the anatomical proximity between the stomach and the spleen [11].

It seems that tumor cells encounter more difficulties in invading the spleen; thus, their dissemination via the lymphatic route is challenging due to the presence of poor lymphatic drainage of the spleen [1,12]. On the other hand, hematogenous tumor invasion of the spleen is difficult due to the angle of emergence of the splenic artery from the celiac trunk as well as the presence of smooth muscle in the splenic capillaries, which ensures the expulsion of stored blood [1]. Perhaps the most important protective factor of the spleen against the appearance of metastases is its significant immune role; the spleen can be compared to a “university” where important immune defense elements are formed and “trained.”

However, it appears that the dissemination of malignant gastric cells to the spleen can occur hematogenously, which is also the most frequent route, or by continuity in the case of tumors located at the level of the greater gastric curvature, and more rarely through lymphatic dissemination. Thus, cases have been cited where splenic secondary tumors were discovered in patients without lymphatic invasion (N0) at the time of performing radical gastrectomy with curative intent [7]. The 6th edition of the Japanese Gastric Cancer Treatment Guidelines (JGCT) also recommends performing a non-standard gastrectomy with extended D2 lymphadenectomy and excision of station 10 with or without spleen preservation only in cases of proximal gastric cancers invading the greater curvature and does not recommend performing this routinely [13]. However, there are studies that have shown that, in addition to invasion of the greater curvature, tumor size as well as advanced tumor stage at diagnosis can be associated with a high risk of invasion of lymph node station 10 [14]. However, no real benefit in terms of survival has been demonstrated for patients who underwent extended lymphadenectomy to station 10; thus, surgical excision of this station is not routinely indicated [14].

Studies have found that male patients are more likely to develop splenic metastases from gastric cancer [12]. These can be synchronous when discovered at the same time as the malignant disease diagnosis or metachronous when diagnosed during the follow-up period of these patients [12]. It also seems that the survival of patients with gastric cancer and synchronous splenic metastases is shorter compared to those with metachronous splenic metastases [2].

In the last 25 years, we have identified only 15 articles in PubMed and GoogleScholar databases that present cases similar to ours. To identify these, we used the following keywords: gastric cancer, gastric tumor, splenic tumor, splenic metastasis, and metachronous tumor, which we used in various combinations. We excluded articles that included cases of gastric cancer with synchronous splenic metastases. The main characteristics of the identified studies, along with our case, are presented in Table 3.

In the case of the patient we presented, the splenic metastatic tumor was discovered approximately 10 months after gastrectomy; however, in the literature, this interval can vary from 2 months to 8 years [12].

On the other hand, splenic metastases are found in the advanced stages of malignant gastric disease in the context of multiorgan secondary determinations, but the literature also cites cases of early gastric cancer where splenic metastases were diagnosed more than a year after the endoscopic mucosal resection (EMR) of a lesion at the level of the cardia [15].

A particularity of the presented case is also the antral location of the primary gastric tumor, as splenic metastases are usually more frequent in proximal gastric tumor locations and at the level of the greater curvature.

Since over half of patients with gastric cancer and splenic metastases do not present relevant clinical symptoms, it is important to dynamically determine the values of the tumor markers CEA and CA 19-9, with their increase being confirmed in dynamics [6,7]. On the other hand, there are new markers that seem to play an important role in the prognosis of gastric cancer, namely CD 276 and DKK 3.

CD276 is a transmembrane glycoprotein that is part of the B7 protein family, which was first discovered in 2001 [16]. Its expression is low in normal tissues and high in tumor tissues, and elevated serum levels of it correlate with a negative prognosis for patients with gastric cancer [8,17].

DKK 3 is part of the DKK protein family, which plays a role in the negative regulation of the Wnt signaling pathway, apparently having an antiproliferative role in gastric tumors and beyond [18,19]. Thus, low levels of DKK 3 correlate with advanced gastric malignancy and a lower survival rate [20].

The role of these markers in the postoperative follow-up of gastric cancer patients for assessing tumor recurrence or metastasis has not yet been studied. In our case, we observed a slight increase in CD 276 and a decrease in DKK3; however, these findings cannot be considered significant in the context of a single clinical case but may represent a new direction for research.

On the other hand, a contrast-enhanced CT examination in patients with suspected splenic metastases provides important diagnostic information; however, performing a PET-CT with 18F-FDG highlights the increased metabolism at the level of the splenic tumor and increases the chances of diagnosis. According to studies, the 18F-FDG PET-CT examination seems to have the highest negative predictive value for malignancy in the case of splenic tumors discovered through other imaging investigations [21]. Confirmation of the diagnosis through ultrasound-guided fine needle biopsy of the spleen is feasible, showing a sensitivity of 98.4% and a positive predictive value of 99.2%, with major post-procedure complications being under 1% [22,23].

An important step in the management of these cases is also the differential diagnosis of splenic tumors. Besides benign tumors, various types of lymphomas, or other malignant tumors, a challenging differential diagnosis for splenic metastases of gastric cancer is splenic sarcoidosis, which is more frequently encountered synchronously with the gastric tumor and much less often metachronously after surgical treatment of gastric cancer [24,25]. The CT appearance as well as the intense uptake of 18F-FDG on a PET-CT examination make it difficult to differentiate sarcoidosis from a metachronous splenic metastasis, and sometimes the diagnosis is made based on the histopathological examination of the splenectomy specimen [24,26]. Therefore, performing a preoperative splenic biopsy can avoid the need for a splenectomy in a patient with a history of gastric cancer who presents with high surgical and biological risk.

Until now, there is no standard protocol for the diagnostic and therapeutic management of gastric cancer patients with metachronous splenic metastases, because such cases are rarely cited in the literature. We believe the first important step is establishing the diagnosis of splenic metastasis. Thus, in the case of discovering a splenic tumor formation during the follow-up of patients with a history of gastric cancer, we recommend performing an 18F-FDG PET-CT. This imaging investigation also allows for the exclusion of other systemic metastases. If the spleen is the only location of secondary lesions, splenectomy can be performed on a scheduled basis, along with the completion of the antipneumococcal, antimeningococcal, and anti-Haemophilus influenzae type b vaccination schedule. If there is suspicion of a splenic abscess or splenic sarcoidosis, a fine-needle aspiration biopsy may be performed, the bleeding risk of which is often overestimated [22].

Therapeutic options for gastric cancer patients with metachronous splenic metastases include splenectomy if the spleen is the only secondary site, chemotherapy, and the wait-and-see approach for patients with secondary sites in other organs as well as peritoneal carcinomatosis [11]. There are cases in the literature where, due to the simultaneous presence of peritoneal metastases, chemotherapy with 10 cycles of capecitabine associated with oxaliplatin was chosen, and a complete response was achieved according to RECIST (Response Evaluation Criteria in Solid Tumors) version 1.1 documented by PET-CT with 18F-FDG, which led to a splenectomy. The patient has been free of tumor recurrence for five years after the diagnosis of gastric cancer [11]. Until now, there is no consensus on the follow-up interval for patients who have opted for the wait-and-see method and splenectomy [27].

The minimally invasive approach to splenectomy is the most desirable due to the well-known benefits that it offers the patient, as well as the possibility of evaluating the presence of other secondary peritoneal lesions not visualized preoperatively, in which case the intervention can be limited to exploratory laparoscopy with peritoneal biopsy. While laparoscopic splenectomy is considered the gold standard, robotic splenectomy is gaining ground even in cases of splenomegaly [28,29]. Existing adhesions after gastrectomy can be an impediment to the minimally invasive approach to metachronous splenic metastases of gastric cancer, especially if the gastrectomy was performed through an open approach. In the case we presented, the initial approach to the gastric tumor was laparoscopic, so postoperative adhesion syndrome did not pose significant dissection challenges.

We emphasize that all decisions regarding the management of these patients should be made by a multidisciplinary team in a tumor board that includes, in addition to the oncologist, a surgeon, a radiotherapist, and an infectious disease specialist to implement primary prevention of overwhelming postsplenectomy infection (OPSI), which can occur from one week to 20 years after splenectomy [30]. We must not forget that we are dealing with a vulnerable patient, often immunosuppressed after oncological treatment, but also with absorption and digestion disorders associated with a previous gastrectomy.

**Table 3:** Summary of the identified studies that include cases of gastric cancer with metachronous splenic metastases.

Year First Author Name and References [Ref]	Number of Patients Age and Sex Masculine (M) Feminine (F)	(Gastric) Tumor Characteristics	Tumor Location at Stomach Level	Stage of Gastric Malignant Disease	Time to Metastasis	Splenectomy Treatment: Open (O) Laparoscopic (L) Robotic (R) Unspecified (N)	Adjuvant Chemotherapy Post Splenectomy	Outcomes
2000 Opocher, E. et al. [6]	2 66/76 M/M	gastric adenocarcinoma/poorly differentiated adenocarcinoma	unspecified/lower third of stomach	pT2N1M0/pT2N0M0	36 months/60 months	O	unspecified	no recurrence at 14 months/no recurrence at 13 months
2002 Yamanouchi, K. et al. [31]	1 69 M	gastric adenocarcinoma	distal	unspecified	48 months	O	unspecified	death 40 months postoperatively with multiple metastases.
2008 Sasakawa, H. et al. [32]	1 76 F	moderately differentiated adenocarcinoma	middle stomach body	unspecified	unspecified	N	unspecified	no recurrence at 4 months
2009 Sunitsch, S. et al. [33]	1 80 F	gastric adenocarcinoma	distal	pT2N0M0	36 months	O	unspecified	unspecified
2009 Kawasaki, H. et al. [15]	1 76 M	gastric adenocarcinoma	cardia	early gastric carcinoma	12 months	N	unspecified	no recurrence at 24 months
2011 Deng, Z. et al. [34]	1 54 M	gastric adenocarcinoma	unspecified	unspecified	60 months	N	unspecified	no recurrence at 9 months
2013 Zhu, Y. et al. [2]	1 62 M	poorly differentiated adenocarcinoma	middle third	pStage IIIB	12 months	L	unspecified	no recurrence at 10 months
2013 Kamaleshwaran, K. et al. [35]	1 55 M	gastric adenocarcinoma	fundus and gastroesophageal junction	unspecified	12 months	N	unspecified	unspecified
2014 Santos, M. et al. [7]	1 71 M	well-differentiated adenocarcinoma	distal small curvature gastric	pT3N0M0	72 months	N	unspecified	unspecified
2017 Yoshizawa, J. et al. [12]	1 60 M	poorly differentiated adenocarcinoma	middle third	pT1bN2M0- pStage IIB	12 months	O	No	no recurrence at 18 months
2017 Namikawa, T. et al. [36]	1 75 M	solid-type poorly differentiated adenocarcinoma	upper-third of the stomach	early gastric cancer	28 months	N	unspecified	no recurrence at 2 months
2019 Shimizu, M. et al. [37]	1 69 M	gastric adenocarcinoma	lesser curvature in the upper part of the stomach	T3N0M0 pStage IIA	10 months	N	Yes SOX regimen (S1+ oxaliplatin)	no recurrence at 6 months
2020 Obana, A et al. [27]	1 84 M	solitary metastasis moderately differentiated adenocarcinoma	cardia	pT3N1M0- pStage IIB	8 months	N	No	no recurrence at 5 years
2020 Karakuchi, N. et al. [11]	1 67 M	moderately differentiated adenocarcinoma	middle third	pT4aN2M0-pStage IIIB	24 months	O	chemotherapy before splenectomy, unspecified after splenectomy	no recurrence at 18 months
2022 Tanda, H. et al. [38]	1 77 M	moderately differentiated adenocarcinoma	greater curvature of the stomach in the lower body	pT3N2M0- pStage IIIA	18 months	N	unspecified	no recurrence at 12 months
Our case	1 64 M	moderately differentiated adenocarcinoma	distal to the level of the gastric antrum	pT4aN2M0-pStage IIIB	10 months	L	Yes (S1 + cisplatin)	no recurrence at 12 months

## 4. Conclusions

Despite its anatomical and functional protective mechanisms, the spleen is not an unconquerable fortress for malignant cells, and cases of splenic metastases are cited in the literature, with their number increasing due to advancements in imaging methods for monitoring patients with malignant diseases.

On the other hand, DKK 3 and CD 276 are new markers currently being studied for their prognostic role in gastric cancer and beyond. Although there are currently no studies that confirm their importance in tumor recurrence or the development of metachronous metastases, they could constitute a new direction for research.

Diagnostic and therapeutic decisions for patients with a history of gastric cancer who have metachronous splenic metastases should be made within a multidisciplinary team, taking into account the specific characteristics of each case.

For diagnosis, we consider it very useful to perform a PET-CT with 18F-FDG followed by a preferably minimally invasive splenectomy, which is associated with good long-term survival if the spleen is the only area of metastasis.

## Figures and Tables

**Table 1:** The dynamic variation in CEA (carcinoembryonic antigen) and CA 19-9 (carbohydrate antigen 19-9) values.

Moment of Determination	CEA (Carcinoembryonic Antigen) Normal Values <3 ng/mL for Non-Smokers <5 ng/mL for Smokers	CA19-9 (Carbohydrate Antigen 19-9) Normal Values < 27 U/mL
At the time of diagnosis	6.8 ng/mL	108 U/mL
Post-gastrectomy	3.2 ng/mL	21 U/mL
Before splenectomy	6.5 ng/mL	160 U/mL
After splenectomy	2.9 ng/mL	19 U/mL

**Table 2:** The dynamic variation in DKK 3 (Dickkopf Related Protein 3) and CD 276 (Cluster of Differentiation 276) values.

Moment of Determination	DKK 3 (Dickkopf Related Protein 3) (Sensitivity 0.47 ng/mL, Detection Range 0.78–50 ng/mL)	CD 276 (Cluster of Differentiation 276) (Sensitivity 0.47 ng/mL, Detection Range 0.78–50 ng/mL)
Before gastrectomy	84.646 ng/mL	90.310 ng/mL
Before splenectomy	78.490 ng/mL	94.140 ng/mL

## Data Availability

The original contributions presented in this study are included in the article. Further inquiries can be directed to the corresponding authors.
